# A Review of Teachers' Sentiments and Attitudes in Inclusive Education in China

**DOI:** 10.3389/fpsyg.2021.760115

**Published:** 2021-10-11

**Authors:** Min Yang, Chao Yu

**Affiliations:** ^1^The Engineering Technical College of Chengdu University of Technology, Leshan, China; ^2^Department of Student Affairs, Anhui University of Technology, Ma'anshan, China

**Keywords:** teacher training, inclusive education, attitude, sentiment, Chinese educational government, supportive classroom environment

## Abstract

Students should receive appropriate and comprehensive educational opportunities regardless of their ethnicity, gender, and even probable disabilities or exceptionalities. For this purpose, governments and educational boards have agreed to investigate the concept of inclusive education as a new paradigm where students can benefit from materials and classroom environment whether they are ordinary students or students with special needs. Chinese educational government has also adopted inclusive education within its pedagogic program since the middle of the 1990s. In this regard, some well-known researchers highlighted the impact of teachers' attitudes, sentiments, and concerns in inclusive education as a driving force toward student support and rapport. Moreover, the cultural background has also been emphasized in studies of inclusive education. Hence, it is necessary to employ the proposed and standardized attitude, sentiment, and concern scales, as well as the translated version to measure the factors affecting the proper implementation of inclusive pedagogy. The present study was an attempt to review related studies on teachers' attitudes and sentiments, particularly in China. Findings suggest that cultural differences might not necessarily contribute to the successful implementation of inclusive programs; however, pre-service or in-service teachers have demonstrated that higher levels of sentiment (efficacy), as well as positive attitude, can lead to the efficient provision of materials and building a supportive classroom environment for ordinary students and more importantly student with special needs.

## Introduction

Educators and educational systems have always attempted to provide equal and appropriate education to students with special needs. Nevertheless, according to Calgary Board of Health (2008), the concept of inclusion in education started to refer to providing opportunities to all students together, particularly those who belong to minorities, come from poor families, and are vulnerable. Besides, Loreman (1999) and Andrews and Lupart (2000) believed that educating all students impartially and within a shared instructive context is the key to inclusive education.

Loreman et al. (2008) asserted that mutual acceptance among students is regarded as a crucial aspect of inclusion classrooms where students can learn more and experience improved self-efficacy and motivation. Consequently, along with significant educational success among students with exceptionalities, inclusion education has also helped ordinary students improve academically (Demeris et al., 2008).

Teachers' sentiments regarding the special educational needs for target students as well as teachers' positive attitude toward inclusion in education can lead to a better understanding of students' special conditions and provision of more appropriate support (Burke and Sutherland, 2004). Hobbs and Westling (1998) asserted that appropriate training as well as positive experiences with exceptional students can result in teachers' positive attitudes toward inclusion.

In addition, Forlin (2008) argued that it is imperative to design and modify teacher education programs consistent with the advent of the educational inclusion paradigm. For instance, according to Article 24 proposed by United Nation (2006), new teacher training courses should focus on the implementation of proper techniques, materials, and communication strategies tailored at supporting all students, even those with disabilities or special needs.

Inclusion in education has been introduced and developed around the globe for the last 4 decades. Deng and Harris (2008) proposed that inclusion programs emerged in the 1980s in China with a focus on providing mandatory schooling for students with disabilities. Moreover, Yan and Deng (2018) asserted that the Chinese Department of Education introduced the Action Plan for Implementing Inclusive Education in Elementary and Secondary Schools in 2013. Consequently, it is necessary to conduct related studies to determine factors influencing and the role of teachers' attitudes and sentiment toward inclusion education. Hence, the present study aimed at investigating previous studies on inclusion in China to identify strengths and weaknesses as well as to promote them within the Chinese context.

## Theoretical Background

Effective and constant education should be delivered to every child in the community. Regardless of gender, ethnicity, economic status, and disabilities, the students should have the right to be provided with equal schooling (Ainscow et al., 2011). Therefore, the concept of inclusion was proposed as an approach to engage students with disabilities in learning tasks within the classroom setting. As Foreman (2001) argued, inclusive education refers to the collaboration of such students with their classmates (normal students or students with disabilities) in ordinary schools.

Sharma et al. (2015) conducted a seminal study on influential factors in the implementation of inclusion education. They reported that the following factors can affect inclusion programs: context policies, proper employment of resources, social and cultural necessities, family's roles, and revisiting and adapting school activities. Furthermore, some researchers asserted that teacher-related factors can play a significant role in the proper adaptation of inclusion education, e.g., teachers' self-efficacy, concerns, and attitudes (Avramidis and Norwich, 2002; Loreman et al., 2014; Specht, 2016) as well as demographic characteristics, type of disability, and teacher-training courses (De Boer et al., 2011).

In an attempt to develop a valid and reliable scale to measure teachers' sentiment, attitudes, and concerns in the implementation of inclusive education, Loreman et al. (2007) conducted a seminal study and proposed a 19-item scale regarding the perception of teachers about inclusion programs accordingly, i.e., the sentiments, attitudes, and concerns about inclusive education (SACIE) scale. They further proposed that teachers' sentiment can help them deal with classes with students who have disabilities, teachers' attitude is also directly related to the successful implementation of teaching approaches, and finally, teachers' concern stems from their uncertainty about their capabilities and preparedness for inclusion schools.

Jordan et al. (2009) contended that teachers' sentiments and attitudes are the predictors of successful inclusion programs. In addition, Forlin et al. (2009) concluded that the implementation of inclusive pedagogy has led to the development and promotion of more positive attitudes and sentiment as well as reduced concerns among educators.

Following the global movement toward inclusive education and, particularly, the UNESCO declaration on Education for ALL (1990), the Chinese government and educational policies have highlighted the need for the implementation of inclusion pedagogy (Liu and Jiang, 2008). More specifically, Deng and Poon-McBrayer (2004) contended that the initial measures concerning inclusion education in China were established in 1994 after the establishment of the learning in the regular classroom (LRC) program. Consequently, it is essential to design pre-service courses for teachers to raise awareness of features of inclusive education in the Chinese context. The present study aimed to review related studies and key empirical findings are introduced in the following section.

## Empirical Studies

There is a great body of research on inclusion programs as well as the effectiveness of teachers' attitudes and sentiment (efficacy) in this regard. We will discuss related studies in terms of cultural diversity and teachers' characteristics.

### Inclusive Education Across Cultures

According to Webber and Lupart (2011), culture is believed to have an impact on inclusive education in different international contexts. For instance, Sharma et al. (2008) conducted a multi-cultural study highlighting educators' attitudes and concerns as well as sentiment about people with disabilities. They investigated 603 undergraduate teacher training programs (Australia = 245; Hong Kong of China = 182; Canada = 58; Singapore = 93). Then, the authors evaluated teachers' attitudes using attitudes toward inclusive education scale (ATIES), developed by Wilczenski (1992). This scale consists of 16 items measuring participants' attitudes toward different aspects of inclusion: social, physical, academic, and behavioral. As a result, it was concluded that teacher training programs can lead to a significant change in pre-service students' attitudes in all contexts except Singapore.

Moreover, findings of Malinen's 2013 Ph.D. dissertation with regard to Chinese, Finnish, and South African pre-service and in-service teachers demonstrated that teachers' sentiment (efficacy) can be categorized as efficacy in collaboration, efficacy in inclusive instruction, and efficacy in managing behaviors. They also reported that these three factors are significantly correlated. Furthermore, all the participants from different countries indicated that there is a significant relationship between the prior experience of teaching students with special needs and high levels of sentiment in inclusive education.

Forlin et al. (2009) conducted a study on 603 pre-service teachers from Australia (*n* = 270), Singapore (*n* = 93), Canada (*n* = 58), and Hong Kong of China (*n* = 182) teachers. They concluded that there is no significant difference between these participants from different cultural backgrounds in terms of inclusive education attitudes, sentiment, and concerns.

Eventually, Murdaca et al. (2016) investigated four hundred Italian teachers' attitudes, sentiments, and concerns in inclusive education using the SACIE-R scale (proposed by Forlin et al., 2011). They confirmed that findings are consistent with other related researches, including the original study. However, the authors had to remove 4 items from the original scale due to the goodness of fit indices for the Italian context.

### Attitudes, Sentiments, and Other Teacher-Related Factors

Li et al. (2016) attempted to investigate pre-service teachers' attitudes, sentiment, and concern regarding inclusion education. For this purpose, they assessed 424 freshman and sophomore Chinese students using the simplified Chinese version of the Sentiments, Attitudes, and Concerns about Inclusive Education Scale Revised (SCACIE-R) (proposed by Forlin et al., 2011). Li et al. (2016) concluded that there is a significant relationship between pre-service teachers' experience with people with disabilities and their attitudes and sentiment about inclusive education. They further reported that students' self-confidence has an influence, yet insignificant, on their belief regarding the implementation of inclusive education.

It is worth noting that Malinen (2013) reported the different findings among pre-service teachers form different countries. For example, Chinese students demonstrated that school patterns in which they choose to work are associated with their self-efficacy, while students in Finland reported that training is positively related to self-efficacy. Besides, male teachers in Finland showed higher capabilities of dealing with students' unfavorable behavior in the classroom. Finally, older participants could score higher in terms of self-efficacy. In addition, Malinen and Savolainen (2008) investigated a sample of 523 Chinese university students by using questionnaire on their perception of the inclusion of children with disabilities and those students with special needs were assigned into regular classrooms. Their study indicated that (a) the participants' average attitude toward inclusion was slightly negative; (b) Social justice, Meeting the special needs of the pupils with severe disabilities, Quality of education and Teachers' competence, were extracted; (c) the most important background variable that explained the attitudes was the participants' major subject in the University; and (d) the ratings for the best educational environment for a student with a disability varied based on different types and levels of disability.

Forlin et al. (2009) concluded that teachers' age might not play a significant role in their attitudes toward inclusive education. Meanwhile, age could have an effect on teachers' previous knowledge in terms of inclusion. In addition, they claimed that gender also does not make significant changes in teachers' sentiment or concern.

From the discussion aforementioned, it can be seen that following the concept of mainstream education in western societies, China has recently started to embrace inclusive education through the learning in regular classrooms (LRC) model. It was established by the Chinese ministry of education in 1994. As Feng (2010) argued, LRC includes the implementation of inclusive education to benefit students with special needs along with ordinary students in an environment of mutual acceptance. Since learning is a dynamic process associated with learners' characteristics, there is always the need for ongoing assessment of teaching students for the purpose of developing required skills and capabilities accordingly.

## Implications and Suggestions for Future Research

Deng and Poon-McBrayer (2012) claimed that a lack of knowledge and experience among teachers who are supposed to work with students with special needs is inevitable. Such lack of awareness can lead to poor instruction quality, particularly in inclusive education programs. Besides, Xiao (2007) asserted that teachers may not have adequate expertise and enough time to get involved with students with disabilities in the classroom. Therefore, it seems necessary to design and implement teacher education courses or programs on how to teach effectively in a mainstream and inclusive education context. It could also include short-term pre-service and in-service courses to improve teachers' perception and skills in this regard.

Previous cross-cultural studies have concluded that the majority of pre-service teachers show similar trends concerning the impact of attitudes and sentiment on inclusive education practices. For instance, Forlin et al. (2009) concluded that pre-service teachers from Australia, Singapore, Canada, and Hong Kong of China follow similar trends in promoting positive attitudes and sentiment toward inclusive education. Nevertheless, it is recommended to conduct further cross-cultural and also longitudinal studies in order to explore new aspects of teacher training programs and the impact of teachers' characteristics in the successful practice of inclusive education.

## Conclusion

By way of conclusion, the current study mainly explored teachers' attitudes and sentiments, particularly in the educational context of China. Besides, the findings suggest that cultural differences might not necessarily contribute to the successful implementation of inclusive programs; however, pre-service or in-service teachers have demonstrated that higher levels of sentiment (efficacy), as well as positive attitude, can lead to the efficient provision of materials and building a supportive classroom environment for ordinary students and more importantly student with special needs. Pre-service teachers should be provided with intership that can help them to gain the working experience that cannot be acquired from their own textbooks. Only in this doing so can novice teachers enhance their instruction quality.

## Author Contributions

All authors listed have made a substantial, direct and intellectual contribution to the work, and approved it for publication.

## Conflict of Interest

The authors declare that the research was conducted in the absence of any commercial or financial relationships that could be construed as a potential conflict of interest.

## Publisher's Note

All claims expressed in this article are solely those of the authors and do not necessarily represent those of their affiliated organizations, or those of the publisher, the editors and the reviewers. Any product that may be evaluated in this article, or claim that may be made by its manufacturer, is not guaranteed or endorsed by the publisher.

## References

[B1] Ainscow M. Dyson A. Goldrick S. West M. (2011). Developing Equitable Education Systems. Abbingdon: Routledge.

[B2] Andrews J. Lupart J. L. (2000). The Inclusive Classroom: Educating Exceptional Children, 2nd. Scarborough, ON: Nelson Canada.

[B3] Avramidis E. Norwich B. (2002). Teachers' attitudes towards integration / inclusion: a review of the literature. Eur. J. Spec. Needs Educ. 17, 129–147. 10.1080/08856250210129056

[B4] Burke K. Sutherland C. (2004). Attitudes toward inclusion: knowledge vs. experience. Education 125, 163–172.

[B5] Calgary Board of Health (2008). Healthy Diverse Populations. Available online at: http://www.crhahealth.ab.ca (accessed May, 2021). /programs/diversity/diversity_resources/definitions/definitions_main.htm (accessed May, 2021).

[B6] De Boer A. Pijl S. Minnaert A. (2011). Regular primary schoolteachers' attitudes towards inclusive education: a review of the literature. Int. J. Inclus. Educ. 15, 331–353. 10.1080/13603110903030089

[B7] Demeris H. Childs R. A. Jordan A. (2008). The influence of students with special needs included in grade 3 classrooms on the large-scale achievement scores of students with special needs. Can. J. Educ. 30, 609–627. 10.2307/20466655

[B8] Deng M. Harris K. (2008). Meeting the needs of students with disabilities in general education classrooms in China. Teach. Spec. Educ. 31, 195–207. 10.1177/0888406408330631

[B9] Deng M. Poon-McBrayer K. (2012). Reforms and challenges in the era of inclusive education: the case of China. Br. J. Spec. Educ. 39, 117–122. 10.1111/j.1467-8578.2012.00551.x

[B10] Deng M. Poon-McBrayer K. F. (2004). Inclusive education in China: conceptualization and realization. Asia Paci. J. Educ. 24, 143–157. 10.1080/02188791.2004.10600206

[B11] Feng Y. (2010). Teacher Career Motivation and Professional Development in Special and Inclusive Education in China. Rotterdam: Sense Publishers. 10.1163/9789460912757

[B12] Foreman P. J. (2001). Integration and Inclusion in Action. Sydney, NSW: Harcourt Brace & Company.

[B13] Forlin C. (2008). “Education reform for inclusion in the Asia-Pacific region: what about teacher education?” in Reform, Inclusion and Teacher Education, eds C. Forlin, and M. G. J. Lian (New York, NY: Routledge), 83–95. 10.4324/9780203895313

[B14] Forlin C. Earle C. Loreman T. Sharma U. (2011). The sentiments, attitudes, and concerns about inclusive education revised (SACIE-R) scale for measuring pre-service teachers' perceptions about inclusion. Except. Educ. Int. 21, 50–65. 10.5206/eei.v21i3.7682

[B15] Forlin C. Loreman T. Sharma U. Earle C. (2009). Demographic differences in changing pre-service teachers' attitudes, sentiments and concerns about inclusive education. Int. J. Inclus. Educ. 13, 195–209. 10.1080/13603110701365356

[B16] Hobbs T. Westling D. L. (1998). Promoting successful inclusion through collaborative problem solving. Teach. Except. Child. 31, 12–19. 10.1177/004005999803100102

[B17] Jordan A. Schwartz E. McGhie-Richmond D. (2009). Preparing teachers for inclusive classrooms. Teach. Teach. Educ. 25, 535–542. 10.1016/j.tate.2009.02.010

[B18] Li X. Xu S. Xiang Y. Potmesil M. (2016). Pre-service teachers' sentiments, attitudes, and concerns about inclusive education in Chongqing, China. E-Pedagogium 16, 7–16. 10.5507/epd.2016.022

[B19] Liu C. Jiang Q. (2008). An Introduction to Special Education. Shanghai: Huadong Normal University Press.

[B20] Loreman T. (1999). Integration: coming from the outside. Interaction 13, 21–23.

[B21] Loreman T. Earle C. Sharma U. Forlin C. (2007). The development of an instrument for measuring pre-service teachers' sentiments, attitudes, and concerns about inclusive education. Int. J. Spec. Educ. 22, 150–159.

[B22] Loreman T. Forlin C. Sharma U. (2014). “Measuring indicators of inclusive education: a systematic review of the literature,” in International Perspectives on Inclusive Education: Measuring Inclusive Education, ed C. Forlin (Bingley: Emerald Group Publishing Limited), 165–188. 10.1108/S1479-363620140000003024

[B23] Loreman T. McGhie-Richmond D. Barber J. Lupart J. (2008). Student perspectives on inclusive education: a survey of grade 3-6 children in rural Alberta, Canada. Int. J. Whole School. 5, 1–12.

[B24] Malinen O. P. Savolainen H. (2008). Inclusion in the east: Chinese students' attitudes towards inclusive education. Int. J. Spec. Educ. 23, 101–109. Available online at: https://files.eric.ed.gov/fulltext/EJ833686.pdf

[B25] Malinen P. (2013). Inclusive Education From Teachers' Perspective Examining Pre- and in-Service Teachers' Self-Efficacy and Attitudes in Mainland China. (Unpublished doctoral dissertation), University of Eastern Finland, Joensuu; Kuopio.

[B26] Murdaca A. Oliva P. Costa S. (2016). Evaluating the perception of disability and the inclusive education of teachers: the Italian validation of the SACIE-R (sentiments, attitudes, and concerns about inclusive education – revised scale). Euro. J. Spec. Needs Educ. 33, 148–156. 10.1080/08856257.2016.1267944

[B27] Sharma U. Forlin C. Loreman T. (2008). Impact of training on pre-service teachers' attitudes and concerns about inclusive education and sentiments about persons with disabilities. Disabil. Soc. 23, 773–785. 10.1080/09687590802469271

[B28] Sharma U. Loreman T. Macanawai S. (2015). Factors contributing to the implementation of inclusive education in Pacific Island countries. Int. J. Inclus. Educ. 20, 397–412. 10.1080/13603116.2015.1081636

[B29] Specht J. (2016). Professional learning for inclusion in Canada. J. Res. Spec. Educ. Needs 16, 894–895. 10.1111/1471-3802.1_12347

[B30] United Nation. (2006). Convention on the right of persons with disabilities. Retrieved July 2021 from http://www.un.org/disabilities/convention/conventionfull.shtml

[B31] Webber C. F. Lupart J. (2011). Leading intercultural inclusive schools: An international perspective. Int. Stud. Educ. Admin. 39, 3–18.

[B32] Wilczenski F. L. (1992). Measuring attitudes toward inclusive education. Psychol. Sch. 29, 306–312. 10.1002/1520-6807(199210)29:4<306::AID-PITS2310290403>3.0.CO;2-1

[B33] Xiao F. (2007). The Chinese learning in regular classroom: history, current situation, and prospects. Chin. Educ. Sock. 40, 8–20. 10.2753/CED1061-1932400401

[B34] Yan T. Deng M. (2018). Regular education teachers' concerns on inclusive education in China from the perspective of concerns-based adoption model. Int. J. Inclus. Educ. 23, 384–404. 10.1080/13603116.2018.1435741

